# Exact arithmetic as a tool for convergence assessment of the IRM-CG method

**DOI:** 10.1016/j.heliyon.2020.e03225

**Published:** 2020-01-27

**Authors:** Josip Dvornik, Antonia Jaguljnjak Lazarevic, Damir Lazarevic, Mario Uros

**Affiliations:** a University of Zagreb, Faculty of Civil Engineering, Kaciceva 26, Zagreb, 10000, Croatia; b University of Zagreb, Faculty of Mining, Geology and Petroleum Engineering, Pierottijeva 6, Zagreb, 10000, Croatia

**Keywords:** Applied mathematics, Exact arithmetic, Benchmark, Rounding error, Iterated Ritz Method, Conjugate gradient method

## Abstract

Using exact computer arithmetic, it is possible to determine the (exact) solution of a numerical model without any rounding error. For such purposes, a corresponding system of equations should be exactly defined, either directly or by rationalising the numerically given input data. In the latter case, there is an initial round-off error, but this does not propagate during the solution process. If this system is exactly solved first and then using floating-point arithmetic, the convergence of the numerical method easily follows. As an example, the IRM–CG, which is an alternative to the Conjugate Gradient (CG) method and a special case of the more general Iterated Ritz Method (IRM), is verified. The method is not based on conjugacy; therefore, restarting strategies are not required, while an overrelaxation factor and preconditioning like techniques could be easily adopted. The exact arithmetic approach is introduced by means of a simple example and is then applied to small structural engineering problems. The perturbation of the displacement increment and the different condition numbers of the system matrix are used to check the stability of the algorithm. Interestingly, a large difference in the number of steps between the exact and numerical approaches is detected, even for well-conditioned systems. According to the tests, the IRM-CG may be considered to be stable and useful for not well-posed or well-posed but ill-conditioned models. Because the computer demands and execution time grow enormously with the number of unknowns using this strategy, three possibilities for larger systems are also provided.

## 1 Introduction: The Iterated Ritz Method

The Iterated Ritz Method (IRM) is an iterative approach to solving the symmetric positive definite (SPD) system Ax=b based on successive minimisation of the corresponding energy (the quadratic function)

(1) $$f\left(x\right)=\frac{1}{2}x^{\text{T}}Ax-x^{\text{T}}b$$

inside a small subspace formed at each step [1]. The main strategy is to present solution increments by the Ritz idea:

(2) $$p_{\left(i\right)}=\Phi_{\left(i\right)}a_{\left(i\right)}$$

where $\Phi_{\left(i\right)}=\left[\phi_{1,\left(i\right)}\phi_{2,\left(i\right)}\ldots \phi_{m,\left(i\right)}\right]$ is a matrix of linearly independent coordinate vectors (that form subspace) and $a_{\left(i\right)}$ is the vector of corresponding coefficients. Using this approach, the energy decrement achieved by (2), is also presented in the quadratic form

(3) $$\Delta f\left(a_{\left(i\right)}\right)=\frac{1}{2}a_{\left(i\right)}^{\text{T}}{\bar{A}}_{\left(i\right)}a_{\left(i\right)}-a_{\left(i\right)}^{\text{T}}{\bar{r}}_{\left(i\right)}$$

where ${\bar{A}}_{\left(i\right)}=\Phi_{\left(i\right)}^{\text{T}}A\Phi_{\left(i\right)}$ and ${\bar{r}}_{\left(i\right)}=\Phi_{\left(i\right)}^{\text{T}}r_{\left(i\right)}$ are the SPD generalised (Ritz) matrix and residual vector, both of order m. Minimisation of (3) leads to a system of equations that should be solved at each step:

(4) $${\bar{A}}_{\left(i\right)}a_{\left(i\right)}={\bar{r}}_{\left(i\right)}$$

This is a very small system, because only several coordinate vectors are applied (m≪n). The solution is used to find the increment in (2), and $x_{\left(i+1\right)}=x_{\left(i\right)}+\omega p_{\left(i\right)}$ is subsequently updated.

Coefficient ω∈(0,2) is the relaxation factor, known from the Successive Overrelaxation method, and might improve the convergence. Some optimal ω exists, but they vary for every unknown and solution step. Moreover, the determination is usually more ‘expensive’ than the benefit of possible improvement. Generally, ω may be guessed by intuition or experience, and kept constant during the solution process.

The residual is recursively defined as $r_{\left(i+1\right)}=r_{\left(i\right)}-\omega Ap_{\left(i\right)}$ and should always be corrected after some (say k) number of steps using the equilibrium relation $r_{\left(i+1\right)}=b-Ax_{\left(i+1\right)}$, because of accumulated round-off errors. The process is terminated after the convergence criterion is reached, i.e. $∥r_{\left(i\right)}{∥}_{2}\leq \varepsilon ∥r_{\left(0\right)}{∥}_{2}$, where ε is a very small positive number. To avoid useless calculations (if the algorithm has trouble converging) the maximum number of steps ($n_{max}$) should also be defined. Simple pseudocode, with sequence of instructions common for iterative solution methods, is given by the Algorithm 1.

Therefore, at each step, coordinate vectors spanning the subspace are created, within which the energy of the system is reduced. This is why the small system (4) needs to be solved (most often by some direct solver). If ω=1 it is the largest reduction (local energy minimum), which is not necessarily optimal for global convergence. The procedure leads to a gradient class solution method that combines iterative and direct solution strategies. If the iterative process is convergent, the sum of small-system solutions approaches the large (original) system solution, and the sum of small-system energies monotonically decreases and approaches the minimum of the large system [2].

For the convergence of the IRM one coordinate vector not orthogonal to the current residual is sufficient. It can be $r_{\left(i+1\right)}$ itself, or multiplied by some SPD matrix. A previous solution increment $p_{\left(i\right)}$ contributes to faster convergence, and it is also frequently used. This vector is known from the previous step, therefore it ‘costs’ nothing, contrary to other vectors that should be generated somehow. Such vectors are of different efficiency, but are very freely selected – they should only be linearly independent.

The main difficulty is that the number of potentially good coordinate vectors is very large. Unfortunately, adequate criteria for the selection of generally efficient vectors is not known and the background theory is not well developed. For one group of models some vectors work fine but respond badly for another. Under such conditions, an efficient (small, fast and model independent) subspace would be of great significance. Obviously, a novel and promising iterative solver is established, but further theoretical and practical research is needed.

The IRM can also be considered as a generalisation of some iterative methods [3]. Depending on the choice of coordinate vectors, solvers can be represented or interpreted as special cases of this approach. Furthermore, it is possible to combine good properties of several methods simultaneously. If appropriate vectors are selected, convergence should proceed faster than using any single method considered. Here, an improved conjugate gradient (CG) algorithm (named the IRM-CG) is briefly presented [4], which is applicable to sparse and large SPD systems arising in many physics applications [5, 6, 7, 8].

Algorithm 1 The basic IRM algorithm.

**Table:** 

Require:$\;A,\;b,\;x_{\left(0\right)},\;\omega ,\;\varepsilon ,\;k,\;n_{max}$ {usually $x_{\left(0\right)}\leftarrow 0$}
Ensure: $x_{\left(i+1\right)}$ {close to x**}**
1: i←0 {initialisation: the Steepest Descent}
2: $r_{\left(0\right)}\leftarrow b-Ax_{\left(0\right)}$ {the initial residual}
3: $q\leftarrow r_{\left(0\right)}^{T}r_{\left(0\right)}/(r_{\left(0\right)}^{T}Ar_{\left(0\right)})$ {the initial step length}
4: $p_{\left(0\right)}\leftarrow qr_{\left(0\right)}$ {the initial solution increment}
5: **while**$\left(∥r_{\left(i\right)}{∥}_{2}>\varepsilon ∥r_{\left(0\right)}{∥}_{2})\wedge (i\leq n_{max}\right)$**do** {the Iterated Ritz method}
6: generate $\left[\phi_{1,\left(i\right)}\phi_{2,\left(i\right)}\;\ldots \phi_{m,\left(i\right)}\right]$ {define $\Phi_{\left(i\right)}$ by columns}
7: ${{\bar{A}}_{\left(i\right)}\leftarrow \left[\phi_{1,\left(i\right)}\phi_{2,\left(i\right)}\;\ldots \phi_{m,\left(i\right)}\right]}^{T}A\left[\phi_{1,\left(i\right)}\;\phi_{2,\left(i\right)}\;\ldots \;\phi_{m,\left(i\right)}\right]$ {define ${\bar{A}}_{\left(i\right)}=\Phi_{\left(i\right)}^{T}A\Phi_{\left(i\right)}$}
8: ${{\bar{r}}_{\left(i\right)}\leftarrow \left[\phi_{1,\left(i\right)}\phi_{2,\left(i\right)}\;\ldots \phi_{m,\left(i\right)}\right]}^{T}r_{\left(i\right)}$ {define ${\bar{r}}_{\left(i\right)}=\Phi_{\left(i\right)}^{T}r_{\left(i\right)}$}
9: $a_{\left(i\right)}\leftarrow {\bar{A}}_{\left(i\right)}^{-1}{\bar{r}}_{\left(i\right)}$ {solve the small system ${\bar{A}}_{\left(i\right)}a_{\left(i\right)}={\bar{r}}_{\left(i\right)}$}
10: $p_{\left(i\right)}\leftarrow \left[\phi_{1,\left(i\right)}\;\phi_{2,\left(i\right)}\;\ldots \;\phi_{m,\left(i\right)}\right]a_{\left(i\right)}$ {calculate $p_{\left(i\right)}=\Phi_{\left(i\right)}a_{\left(i\right)}$}
11: $x_{\left(i+1\right)}\leftarrow x_{\left(i\right)}+\omega p_{\left(i\right)}$ {solution update}
12: **if**imod k≠0**then** {update residual every k steps}
13: $\;\;\;r_{\left(i+1\right)}\leftarrow r_{\left(i\right)}-\omega Ap_{\left(i\right)}$ { from the recursive relation }
14: **else**
15: $\;\;\;r_{\left(i+1\right)}\leftarrow b-Ax_{\left(i+1\right)}$ { from the equilibrium equation }
16: **end if** {end of residual update}
17: i←i+1 { update the step counter }
18: **end while** {end of the Iterated Ritz method}

## 2 Non-recursive CG-like algorithm without the need to restart

The IRM-CG also starts with the Steepest Descent step. Other steps are executed using a simple alternative to the CG simulated by the IRM with two coordinate vectors: $r_{\left(i+1\right)}$ and $p_{\left(i\right)}$. Vectors span a two-dimensional subspace. At each step, a system of two equations is solved and a local energy minimum within that plane is found (Algorithm 2). As in the CG, only one matrix-vector multiplication is required per step (line 13, Algorithm 2), with appropriate transformations.

Local energy minima are numerically ‘exact’, contrary to the standard CG where the solution of two equations is sought by the equivalent recursive A-orthogonalisation. Because of the accumulation of round-off errors, orthogonality only really exists for a few adjacent vectors and convergence difficulties are present for large ill-conditioned systems. Many restart [9] and preconditioning techniques [10, 11, 12, 13, 14] improve convergence.

In the IRM-CG, error in A-orthogonality also exists, but orthogonality is not used nor accumulated during an iterative process. Therefore, inherited errors decrease, though non-exact arithmetic (as in every numerical process) affects (but does not threaten) the convergence. As a consequence, restarting of the IRM-CG is not needed. Further, preconditioning-like techniques [15] can be adopted easily [4].

One more advantage of this formulation is natural adoption of the relaxation factor. Using ω≠1, A-orthogonality is lost, which contradicts the standard CG algorithm, which is just based on the A-orthogonality. Therefore, only preconditioners of the classical CG could be modified by ω.

## 3 The IRM-CG is equivalent to the CG

These methods are equivalent, and it is possible for them to be interchanged [4]. Each step may be performed by the CG or the IRM-CG, regardless of how the earlier steps were realised. If the CG is preferred, we suggest that a single IRM-CG step is occasionally executed, before the orthogonality error becomes too large. This could be termed ‘refresh’ rather than ‘restart’.

If exact arithmetic is considered, the IRM-CG and the CG have an identical sequence of step results. The exact solution is obtained after the total number of m steps, where m is the number of different ‘active’ eigenvalues [16]. If b is represented as a sum of eigenvectors $v_{j}$, i.e. $b=\sum^{}a_{j}v_{j}$, eigenvectors (and corresponding eigenvalues) with $a_{j}\neq 0$ are ‘active’ (‘inactive’ otherwise). Of course, m can be found only if all n eigenpairs are detected. Multiple eigenvalues should be counted as one, and ‘inactive’ eigenvalues are not counted at all. This comment is of less practical significance, because the IRM-CG is interesting as an iterative, not as a direct solution method [17]. It can be concluded that the IRM-CG (and the CG) can be used in the study of linear switched systems [18].

## 4 Simple illustrative example

**Figure 1:**
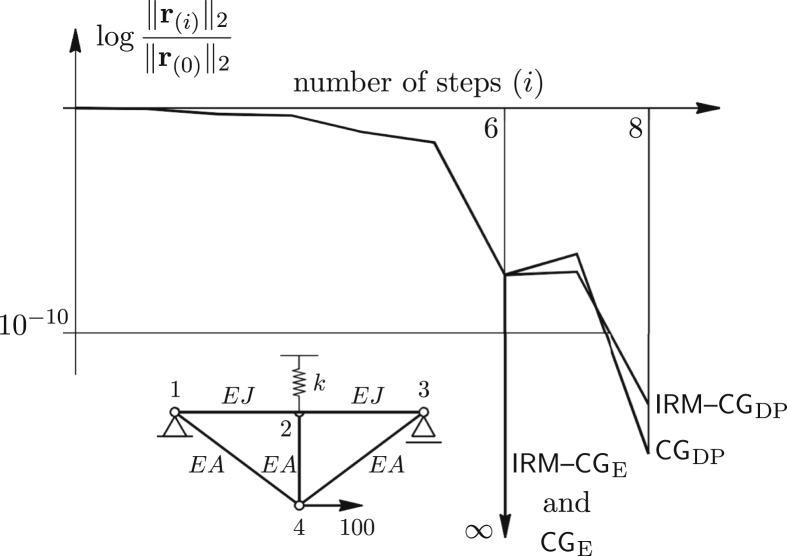
Relative residual norms of the exact (E) and double-precision (DP) implementation of the IRM–CG and the CG applied to simple structural system.

The above considerations are easily proved by the exact arithmetic approach [4], which is extended to the linear structural models that are exactly defined by the direct stiffness method. This is demonstrated by the simple example (Figure 1). Elements of A and b are rational numbers and integers. A system of equations is solved by the CG and the IRM-CG with the exact arithmetic (subscript E).

Algorithm 2 The basic IRM-CG algorithm.

**Table:** 

**Require**: $A,\;b,\;x_{\left(0\right)},\;\omega ,\;\varepsilon ,\;k,\;n_{max}$ {usually $x_{\left(0\right)}\leftarrow 0$}
**Ensure**: $x_{\left(i+1\right)}$ {close to x**}**
1: i←0 {initialisation: the Steepest Descent}
2: $r_{\left(0\right)}\leftarrow b-Ax_{\left(0\right)}$ {the initial residual}
3: $q\leftarrow r_{\left(0\right)}^{T}r_{\left(0\right)}/(r_{\left(0\right)}^{T}Ar_{\left(0\right)})$ {the initial step length}
4: $p_{\left(0\right)}\leftarrow qr_{\left(0\right)}$ {the initial solution increment}
5: $\beta_{\left(0\right)}\leftarrow Ap_{\left(0\right)}$ {the new initialisation}
6: **while**$\left(∥r_{\left(i\right)}{∥}_{2}>\varepsilon ∥r_{\left(0\right)}{∥}_{2})\wedge (i\leq n_{max}\right)$**do** {the IRM-CG method}
7: $x_{\left(i+1\right)}\leftarrow x_{\left(i\right)}+\omega p_{\left(i\right)}$ {solution update}
8: **if**imod k≠0**then** {update residual every k steps}
9: $\;\;\;r_{\left(i+1\right)}\leftarrow r_{\left(i\right)}-\omega \beta_{\left(i\right)}$ {from the recursive relation}
10: **else**
11: $\;\;\;r_{\left(i+1\right)}\leftarrow b-Ax_{\left(i+1\right)}$ {from the equilibrium equation}
12: **end if** {end of residual update}
13: $\alpha_{\left(i\right)}\leftarrow Ar_{\left(i+1\right)}$ {sole matrix-vector multiplication}
14: ${{\bar{A}}_{\left(i\right)}\leftarrow \left[r_{\left(i+1\right)}\;\;p_{\left(i\right)}\right]}^{T}\left[\alpha_{\left(i\right)}\;\;\beta_{\left(i\right)}\right]\;$ {${\bar{A}}_{\left(i\right)}$is symmetric: $r_{\left(i+1\right)}^{T}\beta_{\left(i\right)}=p_{\left(i\right)}^{T}\alpha_{\left(i\right)}$}
15: ${{\bar{r}}_{\left(i\right)}\leftarrow \left[r_{\left(i+1\right)}^{T}r_{\left(i+1\right)}\;\omega r_{\left(i+1\right)}^{T}p_{\left(i\right)}\right]}^{T}$ {if ω=1 the second term is zero}
16: $a_{\left(i\right)}\leftarrow {\bar{A}}_{\left(i\right)}^{-1}{\bar{r}}_{\left(i\right)}$ {solve the small system ${\bar{A}}_{\left(i\right)}a_{\left(i\right)}={\bar{r}}_{\left(i\right)}$}
17: $p_{\left(i+1\right)}\leftarrow \left[r_{\left(i+1\right)}\;\;p_{\left(i\right)}\right]a_{\left(i\right)}$ {update the solution increment}
18: $\beta_{\left(i+1\right)}\leftarrow \left[\alpha_{\left(i\right)}\;\beta_{\left(i\right)}\right]a_{\left(i\right)}$ {update β}
19: i←i+1 {update the step counter}
20: **end while** {end of the IRM-CG method}

In numerical computation, the set of real numbers is in fact approximated by the set of rational numbers represented to a fixed number of decimal digits chosen in advance [19, 20]. On the contrary, by using the exact arithmetic approach we always manipulate with the whole numbers, fractions, constants like π and e, the nth root of such numbers and so on. Generally, we operate with symbolic expressions. Compared to numerical computation, this is a demanding strategy which may result in very complicated and lengthy output, but it is not affected by the rounding error. Such error is completely avoided.

Both algorithms are realised with the Wolfram Language [21] used in the Wolfram Mathematica, version 11.3 [22]. Briefly, if all input data are given as fractions and integers, the Wolfram Language retains the rational arithmetic during execution. Results at every step, (such as residuals, displacements, reactions or internal forces) are also exact and are the same by both methods. The system has 8 DoF and 6 active eigenvectors, because $a_{3}$ and $a_{7}$ are zero (as an integer). Therefore, six steps are needed to obtain the solution. After initialisation and $∥r_{\left(0\right)}{∥}_{2}=1$, remaining relative residual norms are:

(5) $$\frac{1}{2}\text{, }3\frac{\sqrt{15881}}{814}\text{, }48\frac{\sqrt{1134618045}}{20474189}\text{, }24\frac{\sqrt{\cdots }}{\cdots }\text{, }2306892210264\frac{\sqrt{\cdots }}{\cdots }\text{, }0$$

The fourth and the fifth norm contain very large integers (marked with dots) and are not included here. The sixth norm is exact zero. Final displacement components are

(6) $$x_{\left(6\right)}=\frac{1}{a}{\left[\begin{array}{cccccccc} 1440 & 11440004167 & 3840 & 0 & 22880008334 & -1440 & \frac{\cdots }{64} & \ldots \end{array}\right]}^{\text{T}}$$

where a=39440013077. The last two values are also too large to show them here. It is worth noting that, because of the beam symmetry condition, the fourth DoF (rotation of the second joint) is also the integer zero. Even for a such small system precision deteriorates, if a double-precision arithmetic is used (subscript DP). Therefore, the solution is found after two more (eight) steps, providing $\varepsilon \leq 10^{-10}$ is satisified (see Figure 1).

## 5 Check of the algorithm stability

For such a small example, an exact check of the algorithm stability is possible. Because the number of operations is small and the rounding error is negligible, both methods are intentionally perturbed by δ=1[4], added (for example) to the seventh component of $p_{\left(1\right)}$ (at the end of the first step):

(7) $$p_{7,\left(1\right)}\leftarrow p_{7,\left(1\right)}+\delta$$

**Figure 2:**
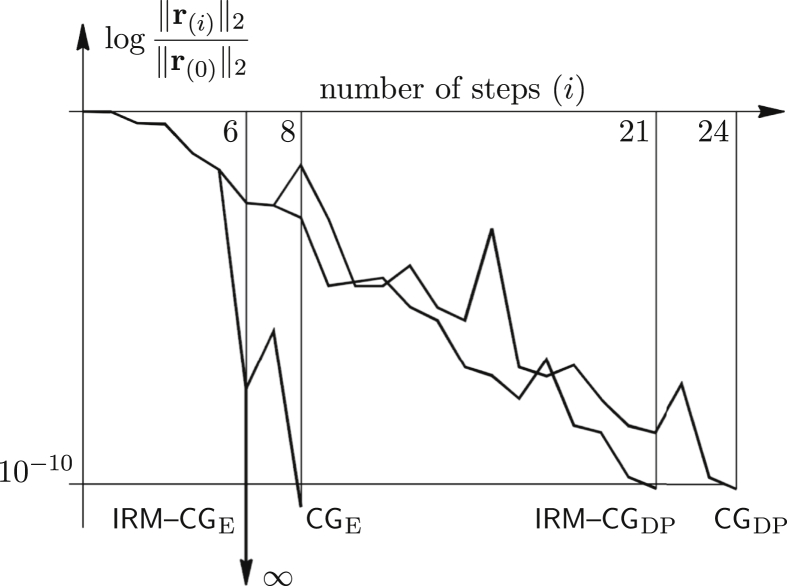
Previous example solved similarly, but with the perturbation of$p_{7,\left(1\right)}$.

This effect is similar to the loss of orthogonality, which is common for the iterative methods, if applied to large and ill-conditioned systems. Then, exact arithmetic is again used to obtain the solution. Even with the perturbation, IRM–CG gives an exact result, because δ is in the loading (residual) direction and therefore lies in the plane spanned by the coordinate vectors. In this particular case, the behaviour of the IRM-CG is as if δ=0 (Figure 2).

Roughly, if δ is split into two components at each step, the one inside, and the other orthogonal to the plane, the first component is exactly resolved and does not produce inherited error. Using CG, both components cause propagation of error and two more steps are needed to obtain the solution. Similar behaviour is noticed in the double-precision environment (added to Figure 2).

Such perturbations may be ‘induced’ by the program code of any solution method. Convergence is then verified by comparing the results obtained with exact and floating point implementation.

## 6 More general examples

**Figure 3:**
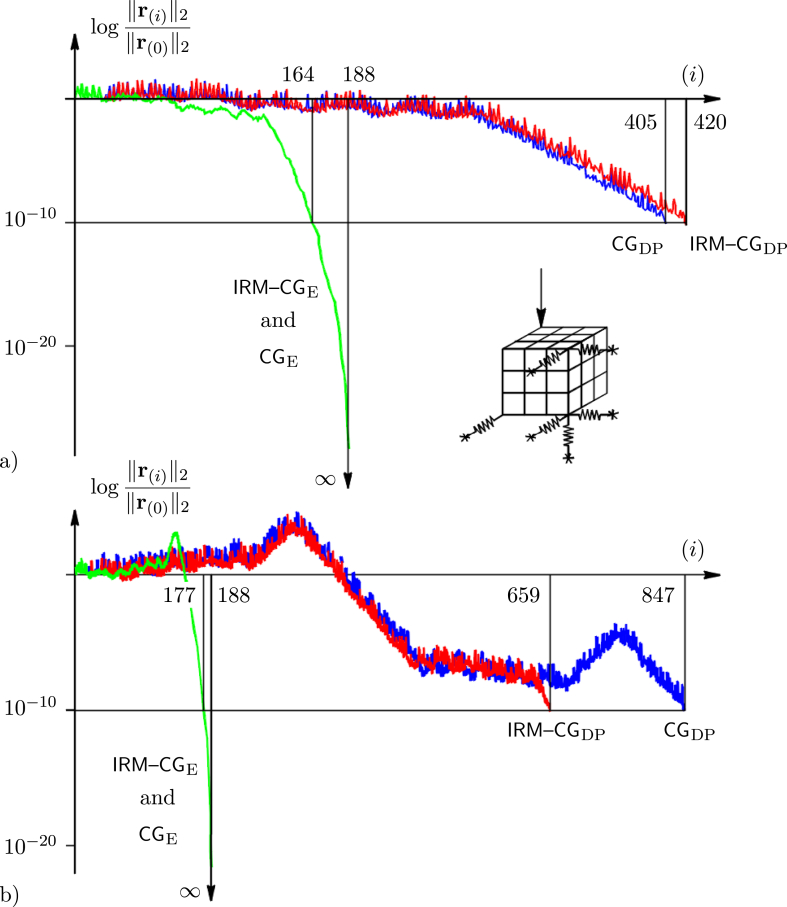
Relative residual norms of the exact and double-precision implementation of the CG and the IRM–CG applied to the finite element analysis of cube model: a) $\kappa \left(A\right)=7.7\cdot 10^{5}$, b).$\kappa \left(A\right)=6.4\cdot 10^{12}$.

Consider a larger model – minimally supported (externally statically determinate) cube, loaded with the unit force at the top (Figure 3a). The cube is discretised by a single Lagrangian $C^{0}$ finite element with 192 DoF. Inner nodes are not statically condensed (but without loss of generality they can be) and the stiffness matrix is resolved exactly, using rational numbers [23]. Corner stiffnesses are defined similarly and are used to control the condition number κ(A), calculated as the ratio of the extreme eigenvalues.

Regardless of κ(A), exact relative residual norms are the same for both methods, and the processes stop after m=188 steps. This is because four inactive eigenvalues (eigenvectors orthogonal to the loading) are detected. The last residual norm is integer zero, $∥r_{\left(188\right)}{∥}_{2}=0$, which means that exact solution of a numerical model is obtained, $x_{\left(188\right)}=x$. Just for curiosity's sake, vertical deflection of the loaded point (last, 192th component of vector x is:

(8) $$x_{192,\left(188\right)}=-\frac{644640386824103250664532957920437457759325413}{288744303487566466737409740217179460197860000}$$

In the numerical environment, methods respond similarly to a well-conditioned model (Figure 3a), but for the ill-conditioned case the IRM–CG is more stable, especially if higher accuracy ($\varepsilon =10^{-10}$) is needed (Figure 3b). Of course, approximate results (for example $x_{\left(405\right)}$ and $x_{\left(420\right)}$, or $x_{\left(659\right)}$ and $x_{\left(847\right)}$) are mutually close and match the exact solution $x_{\left(188\right)}$ reasonably well.

This strategy can also be applied to more complex models (not only) from structural engineering practice, in a combination of various finite elements. If a stiffness matrix is not exactly defined, it is always possible to be rationalised. Elements that are very close to zero could be replaced by the exact integer zero. Using this strategy, the initial roundoff error remains, but it is not accumulated during the solution process (providing exact arithmetic is used). The result is very close to the exact solution of a numerical model $x_{\left(m\right)}\approx x$. In other words, using rationalisation a slightly different model is obtained, but it can be solved exactly. Again, detailed algorithm performance (various steps and final results) can be compared with that obtained by the floating point arithmetic, executed with various numbers of significant digits.

**Figure 4:**
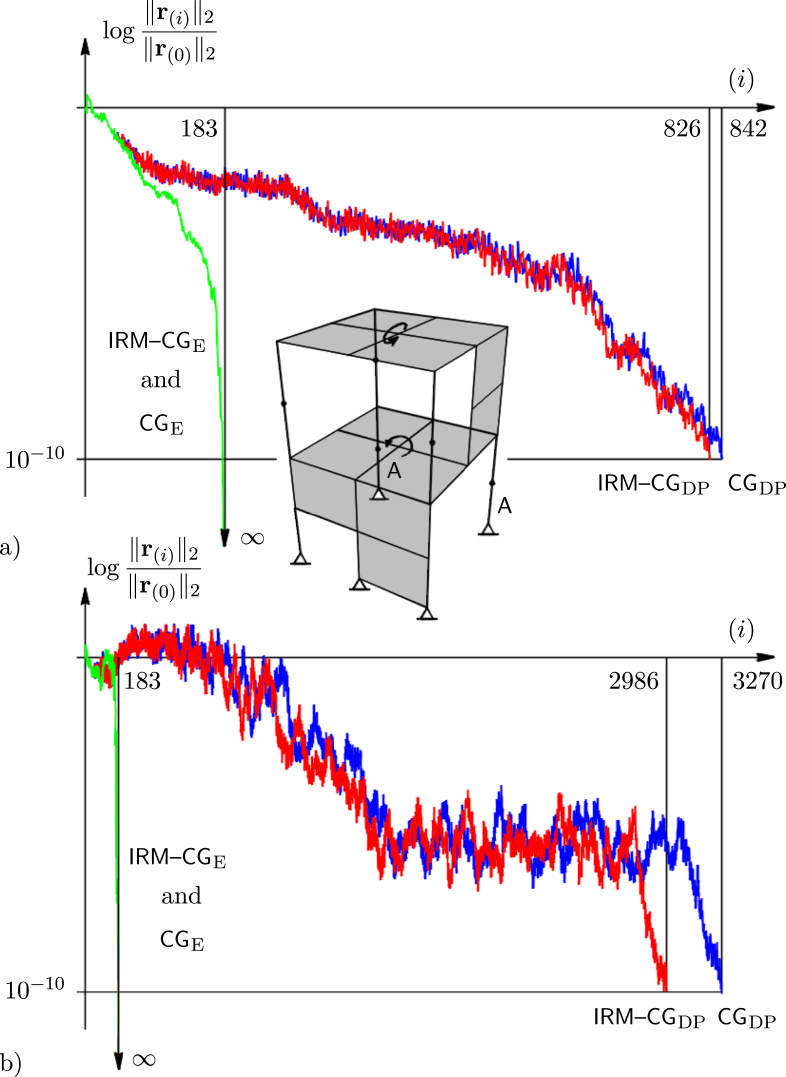
Relative residual norms of the exact and double-precision implementation of the CG and the IRM – CG applied to the finite element analysis of two-story structure: a) $\kappa \left(A\right)=1.1\cdot 10^{5}$, b).$\kappa \left(A\right)=7.9\cdot 10^{8}$.

For example, the model from Figure 4 comprises beam and thin shell elements with displacements and rotations as unknowns. The system has pinned supports and is loaded by two unit moments applied at the centres of the plates. Here, the condition number is controlled by changing the stiffnesses of two corner columns denoted by A. The system has 183 unknowns and all eigenvectors are active (all $a_{j}\neq 0$, thus m=n). Therefore, using exact arithmetic, the solution is found after 183 steps (Figures 4a and 4b). As in the previous example, the IRM–CG is better for the non-well-posed problem, if higher accuracy hasto be achieved.

Interestingly, there is a large difference in the number of steps between the exact and double-precision approach, even for a well-conditioned systems. Curves are mutually close only at the early stage of calculation (at the very beginning they practically collide), because rounding error is not accumulated enough.

Obviously, an increase of the accuracy to more than 16 decimal digits of mantissa may be justified. With more significant figures, residual curves that correspond to the numerical implementation of methods are closer to each other, and converge to the curve of the exact approach. In the limiting case (theoretically, for an infinite number of digits), all three curves must collide. For reasons of clarity, residual functions obtained by the higher precision arithmetic are not added to the figures.

**Figure 5:**
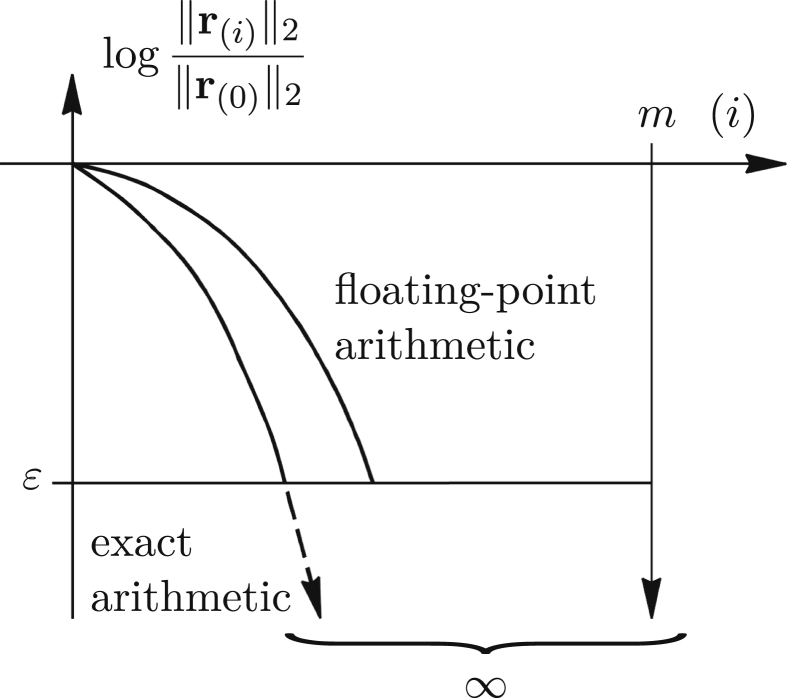
Typical decrease of the relative residual norms for large system.

It should be emphasized that, albeit more realistic, small systems are analysed herein. If the results from Figures 3 or 4 were to be valid for a large system, the methods would be inefficient. In practice, it is just the opposite – the number of steps is much smaller than the number of unknowns. An explanation is given in Figure 5, where a typical decrease of the residual norm for a large number of DoFs (active eigenvaluesm) is sketched.

If we look at the energy (hyper)ellipsoids, the exact CG path to the minimum (solution point) usually consists of smaller number of steps than the path that is obtained by the numerical approaches. However, in very rare situations, rounding errors may be beneficial and direct the path more towards the subspace spanned by only several ellipsoid axes, or push it closer to the just one axis, or much better, near the minimum point, making the number of steps even smaller. In such exceptional cases, the residual curve corresponding to the floating-point arithmetic lies below the curve of the exact approach. Furthermore, in that scenario, round-off errors are not highly accumulated, and they rather very often cancel each other out during the solution process [24].

**Figure 6:**
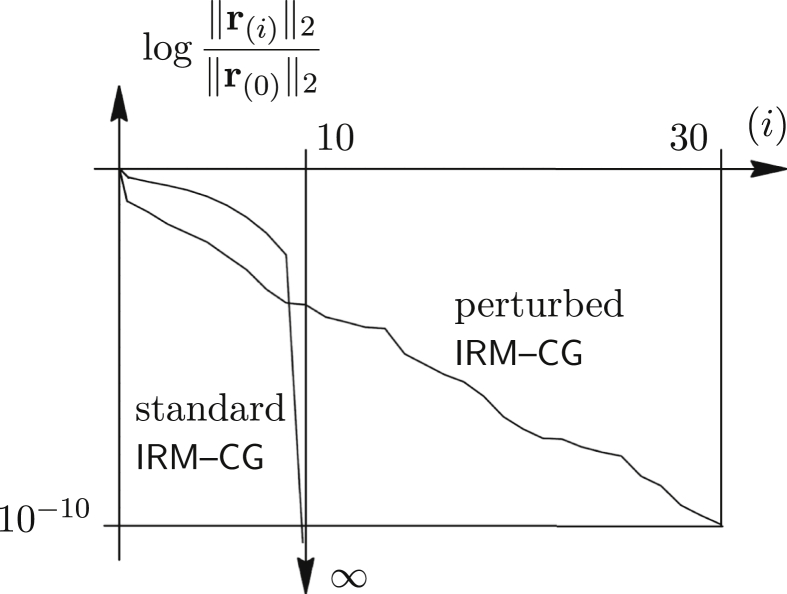
Exact relative residual norms of standard and perturbed IRM–CG.

Consider a diagonal system with $a_{j,j}=j-1/2$ and $b_{j}=1$ (j=1,…,10), which we intend to solve exactly. If the initial perturbation s of the IRM–CG is imposed such that $q\leftarrow sr_{\left(0\right)}/\left(s^{\text{T}}As\right)$ and $p_{\left(0\right)}\leftarrow qs$ (lines 3 and 4 of the Algorithm 2 are modified for that purpose); after some trial and error we found that ${s=\left[\;201\;\;\;\;60\;\;\;\;29\;\;\;\;22\;\;\;\;17\;\;15\;\;\;\;14\;\;\;\;11\;\;\;\;10\;\;\;\;9\;\right]}^{\text{T}}$ directs the solution path more towards the minimum and for i<10 the iterative process behaves better than if such perturbation is missing (Figure 6). Of course, at i=10 the residual curve of the unperturbed process finishes – drops to infinity, because m=10 and $r_{\left(10\right)}=0$ (this is the exact zero vector). For 10<i≤30 the perturbed process continues until $\varepsilon =10^{-10}$ is satisfied.

It should be stressed that if large systems are considered, a large number of operations would be executed and the rounding error may seriously deteriorate the solution, especially if the condition number is large. In the nonlinear environment, a system should be solved multiple times and the number of operations additionally rises. It is not possible to estimate the influence of the rounding error just by checking the results. Furthermore, double precision is not the complete answer to such troubles, as has been frequently said.

## 7 Possibilities for large systems

If a large system of equations needs to be solved, more steps, computer memory, and time are required to obtain (not only) an exact solution. Symbolic expressions become very complicated, while whole numbers and fractions grow enormously. Such problems can be overcome by three strategies.

First, instead of solving exactly, arbitrary-precision arithmetic (with more significant figures than fixed-precision arithmetic) may be applied. In that case, not exact, but very accurate solutions may be found.

Second, an inverse method can be used to establish a benchmark. Simply put, a system matrix is multiplied by some solution to find the corresponding right-hand side vector. The input data and matrix-vector multiplication need to be exact. Such an example is then solved by some numerical (here iterative) method, and the results should be easily compared.

Third, systems with a diagonal matrix may provide an interesting approach. This strategy is based on the consequences of the spectral theorem. Briefly, every SPD matrix can be diagonalised as $V^{\text{T}}AV$, with diagonal elements as eigenvalues and $V=\left[\;v_{1}\;\;\;\;v_{2}\;\;\ldots \;\;v_{n}\;\right]$. The spectra of the original and diagonal matrix are the same. The corresponding right-hand side vector is $V^{\text{T}}b$. This transformation is actually a rotation such that the eigenvectors become parallel to the space coordinate axes. If the original system is solved using the IRM–CG or the CG and steps are then transformed (rotated to that specific position), the results are equal as if the methods had been directly applied to a diagonal system. Therefore, work with a diagonal or original matrix is equivalent (except for the rounding errors if floating-point arithmetic is used).

It should be mentioned that this transformation is based on the eigensolution, which is more ‘expensive’ than the solution of the corresponding system. Therefore, such a strategy (mostly) makes no sense for real-life engineering problems; it could only be used for clever benchmarks and for tests used to analyse various numerical methods.

Furthermore, for such experiments (with either exact or floating-point arithmetic) the above mentioned transformations of the original matrix are not needed. Put simply, an appropriate diagonal matrix is formed directly. Here ‘appropriate’ means that the matrix spectrum, condition number, or eigenvalue distribution are easily controlled and that their effects on solver performance could be clearly recognised. Moreover, even for large systems, memory and time requirements are not very demanding and change of the residual spectrum [2, 3] over steps is easy to follow. Of course, purpose of this approach is not to efficiently solve a system, but to get insight into the behaviour of iterative methods. Solution to a diagonal system is easily obtained directly, because equations are mutually independent.

## 8 Conclusion

With the rapid development of computer algebra systems, exact arithmetic has become a very useful tool for the convergence assessment of numerical solution methods. Herein, a simple equivalent to the CG named the IRM-CG was tested. The method is not based on conjugacy; therefore, it has several advantages over the classical CG.

Its good behaviour is confirmed by analysing three structural engineering examples, which are exactly and numerically solved using both methods. Algorithms are realized using Wolfram Mathematica. Because the methods are equivalent, the exact residual curves always collide and for relatively small condition number they are pretty close. However, for large (and still acceptable) condition number, the results differ in favour of the IRM-CG. Therefore, the method is considered to be very stable, and it should be useful for not well-behaved problems, especially if stronger convergence criterion is adopted. Additionally, even if the CG is preferred as the solution method, it can be restarted using the IRM-CG, which may be called “refresh” instead of “restart”.

Two more things should be mentioned. First, the stiffness matrix and the load vector from the finite element analysis model may always be rationalised. In such cases, a slightly different model is obtained, but it can be exactly solved. This strategy greatly increases the number of problems that can be attacked by this approach. Second, if a large system of equations is considered, three ideas may be exploited: arbitrary precision arithmetic, the inverse solution method and the controlled diagonal system. All can help to overcome the computing demands of the exact arithmetic if larger systems are analysed.

Finally, we start considering a parallel implementation and nonlinear approach to algorithms 1 and 2. Such strategies are extremely useful for large linear and nonlinear models because number of operations and solution time increase rapidly with the number of unknowns. Additionally, the property of conjugacy, which underlies many iterative methods and is valid only for linear models, is not absolutely necessary here. Therefore, we expect that the IRM and the IRM-CG can be very efficient in the nonlinear environment (including optimisation) where conjugacy is not even defined.

Using this methodology, together with the increased computing power, it is possible to establish exact or very precise and more realistic benchmarks for algorithm performance testing.

## Declarations

### Author contribution statementa

J. Dvornik, A. J. Lazarevic: Analyzed and interpreted the data; Wrote the paper.

D. Lazarevic: Conceived and designed the experiments; Performed the experiments.

M. Uros: Contributed reagents, materials, analysis tools or data.

### Funding statement

This work was fully supported by the Croatian Science Foundation under the project IP–2014–09–2899.

### Competing interest statement

The authors declare no conflict of interest.

### Additional information

No additional information is available for this paper.
